# Abdominal Cocoon Syndrome: Two Cases of an Anatomical Abnormality

**DOI:** 10.1155/2019/3276919

**Published:** 2019-04-03

**Authors:** Apostolos Sovatzidis, Eirini Nikolaidou, Anastasios Katsourakis, Iosif Chatzis, George Noussios

**Affiliations:** ^1^Department of Surgery, Agios Dimitrios General Hospital, Thessaloniki 54634, Greece; ^2^School of Physical Education and Sports Sciences of Aristotle University of Thessaloniki, 54623, Greece

## Abstract

**Introduction:**

Idiopathic sclerosing encapsulating peritonitis or abdominal cocoon syndrome (ACS) is a rare anatomical deformity characterized by the partial or complete encasement of the small intestine with fibrotic peritoneum. 193 cases have been described worldwide. The aim of this study is to present two cases of ACS successfully treated at the Surgical Clinic of the Agios Dimitrios General Hospital in Thessaloniki, Greece.

**Presentation of Cases:**

Two men (55 and 54 years old) presented to the emergency department complaining of abdominal pain, distension, constipation, nausea, and vomiting. Neither of these patients had any previous operations. The computed tomography scan of the first patient showed considerable distension of the small bowel, suggestive of internal herniation. The second case showed distention of the jejunum with no obvious cause. Both patients underwent emergency surgery. Intraoperatively, it was found that a fibrous membrane had completely covered the small intestine of the first patient and the jejunum and part of the large intestine of the second patient. Adhesiolysis and partial excision of the membrane were performed in both cases.

**Discussion:**

ACS is a rare cause of small bowel obstruction. Although conservative management with immunosuppressants and steroids has been described, surgical treatment is the gold standard.

**Conclusion:**

Preoperative clinical suspicion of this disease can help determine the diagnosis and protect surgeons from intraoperative “surprises”.

## 1. Introduction

Idiopathic sclerosing encapsulating peritonitis is a rare cause of intestinal obstruction. It was first described in 1908 by Owtschinnikow and it was defined in 1978 by Foo et al. [1]. It is characterized by the partial or complete encasement mainly of the small intestine with the peritoneum, leading to chronic inflammation and fibrosis. This rare clinical entity has been described by various names, including peritonitis chronica fibrosa incapsulata, primary sclerosing peritonitis, and abdominal cocoon syndrome (ACS) [2].

The etiology of ACS remains largely unknown. However, secondary sclerosing encapsulating peritonitis due to previous abdominal surgery, peritonitis, tuberculosis, sarcoidosis, or peritoneal dialysis is more widespread. In a recent systematic review, the authors distinguished a third entity called peritoneal encapsulation [3]. Peritoneal encapsulation was first described by Cleland in 1868 [4], and it is an anatomical anomaly characterized by the presence of an accessory peritoneal membrane. It seems that this membrane derives from the yolk sac peritoneum during the first weeks of embryogenesis [4, 5]. The main difference between this and the other two clinical entities is that there is no inflammatory process in ACS. As a result, the patient is asymptomatic, and the anomaly is an incidental finding [4–7].

ACS is classified into three types according to the extent of membrane encasement. In types I and II, the membrane encloses part or the entire small intestine, respectively. In type III, apart from the small intestine, other organs such as the stomach, colon, and liver are also enclosed [8].

According to a recent systematic review, 193 cases of ACS have been reported worldwide, highlighting the rarity of this syndrome [3]. The majority of the cases were male patients, with an average age of 34.7 years old (standard deviation = 19.2). The countries that reported the greatest number of cases were China, India, Turkey, and Nigeria [3].

Clinically, patients with this syndrome present themselves typically with small bowel obstruction. Nausea, vomiting, anorexia, abdominal pain, and a palpable abdominal mass may also exist in a subacute episode, whereas the acute form exhibits a more dramatic symptomatology [9]. The clinical diagnosis is difficult; however, computed tomography (CT) of the abdomen is very useful because it may show small bowel loops concentrated in the center or one part of the abdomen, encased in a soft tissue density mantle [10–12]. Sometimes, CT is combined with a barium follow-through, which reveals delayed transit of the contrast medium and central clumping of the gut. This is often described as a cauliflower sign or accordion pattern [8]. Although a preoperative diagnosis of this rare syndrome is feasible, most cases are diagnosed incidentally intraoperatively.

With regard to treatment, even though conservative management with immunosuppressants and steroids has been previously described, surgery is the gold standard [13]. The operation involves dissection of the membrane and an extensive adhesiolysis. Resection of the bowel is only necessary when it is nonviable [9]. Overall, the postoperative prognosis is excellent [14].

The aim of this article was to present two cases of ACS that were successfully treated at the Surgical Clinic of the Saint Dimitrios General Hospital in Thessaloniki, Greece. Both cases were concerning middle-aged males with no previous abdominal operations.

## 2. Presentation of Cases

### 2.1. Case 1

A 55-year-old male presented to the emergency department with abdominal pain, abdominal distension, nausea, vomiting, and constipation persisting for two days. The patient stated that he had a history of chronic constipation that resolved spontaneously or with laxatives. He had no history of long-term medication, chronic systemic disease, or surgery.

The physical examination revealed abdominal distension, tenderness, and absence of bowel sounds. The rest of the examination was unremarkable. His vital signs were within normal limits, and there was no clinical evidence of peritonitis.

**Figure 1:**
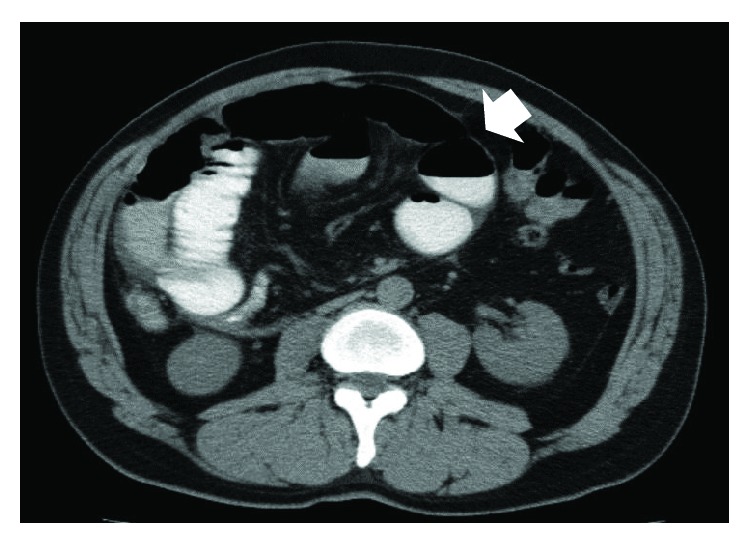
Computed tomography image from case 1. A postoperative second look at the image revealed the presence of a thick fibrotic membrane with a cocoon-like shape that was initially described as internal herniation (arrow).

Abdominal X-rays showed multiple air fluid levels with dilated small bowel loops, suggestive of intestinal obstruction. CT images revealed internal herniation, which occupied part of the right abdomen, containing part of the ileum (Figure 1). The latter appeared distended, likely due to obstruction. The patient also had neutrophilic leukocytosis, and he was taken urgently to theater where an exploratory laparotomy was performed.

**Figure 2:**
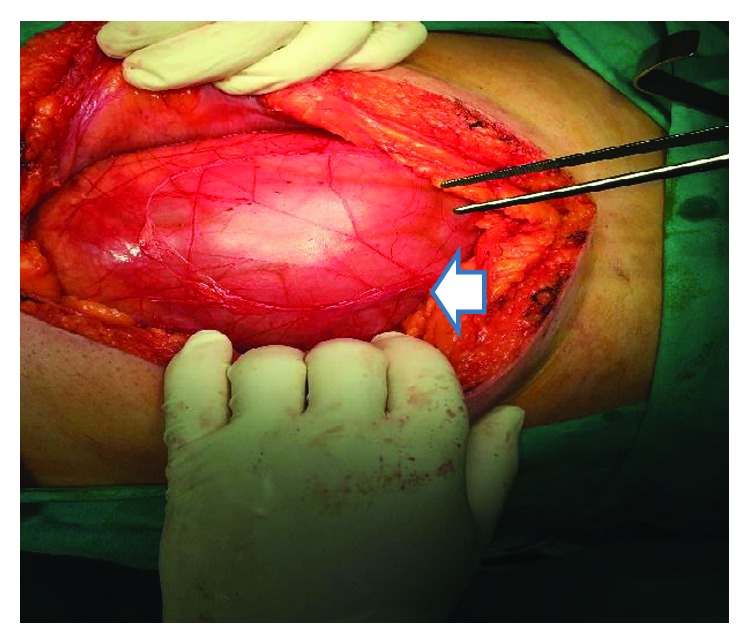
Intraoperative findings of case 1. The membrane covering the dilated loops of the small intestine (arrow).

**Figure 3:**
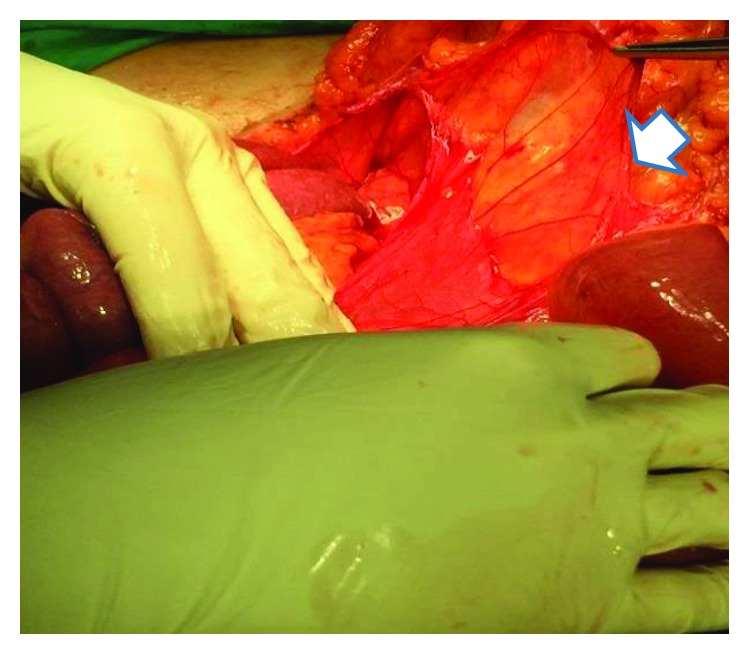
Intraoperative findings of case 1. Intestinal loops released from the cocoon-like membrane (arrow).

Intraoperatively, a fibrotic membrane covering all of the abdominal viscera was found. The small bowel loops were encased and interloop adhesions could be seen (Figures 2 and 3). Incisions were made along the thick membrane in order to release the encased small intestine, and extensive adhesiolysis of the small bowel loops was performed, without resection. The histological findings showed peritoneal fibrosis with sites of chronic nonspecific inflammation. There were no complications during the postoperative period, and the patient was discharged on the 10^th^ postoperative day.

### 2.2. Case 2

A 54-year-old male presented to the emergency department of our hospital with acute cramping abdominal pain and nausea. He had similar episodes during the previous year, with milder symptoms which responded to conservative treatment, without the need for hospitalization. He had no significant past medical or surgical history.

**Figure 4:**
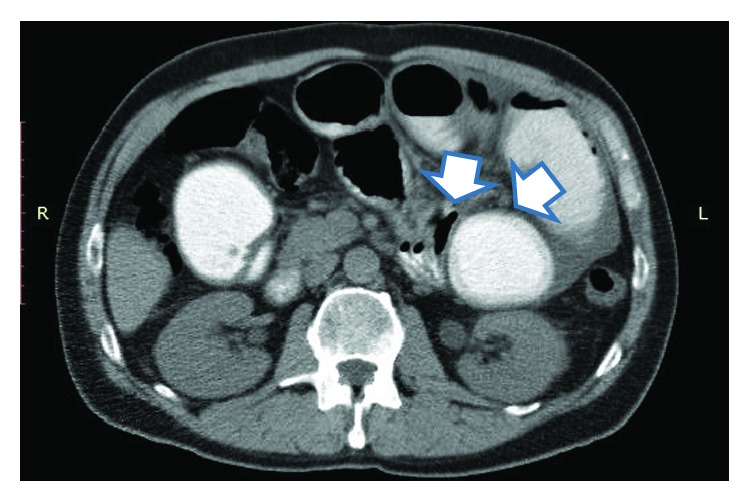
Computed tomography findings of case 2. Dilated small bowel loops, wall thickness, amorphous calcifications, and signs of obstruction could be seen (arrows).

His clinical examination revealed a palpable mass in the right lower paraumbilical area, with sluggish bowel sounds. The radiographic findings were compatible with ileus. Due to the deterioration of his clinical status, a CT scan was performed, which revealed dilatation of the jejunal loops in the left upper abdomen, with fluid collection. Mesentery was thick-walled and amorphous calcifications were also seen (Figure 4). An exploratory laparotomy was planned.

**Figure 5:**
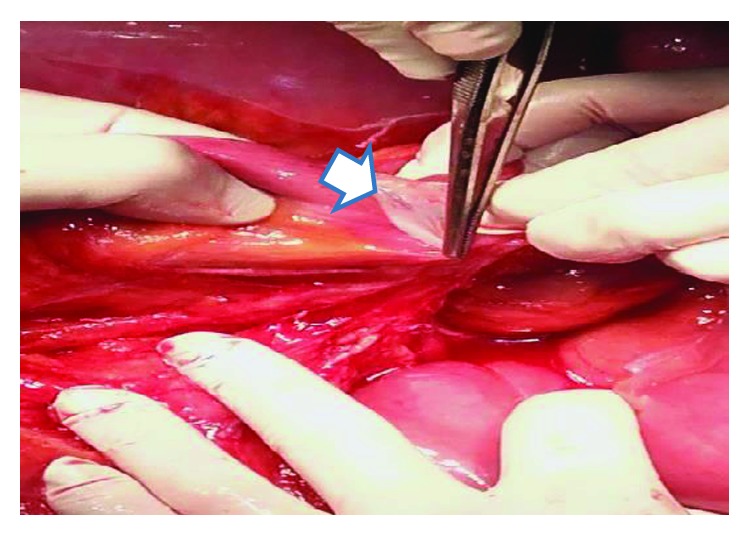
Intraoperative findings of case 2. Excision of the peritoneal membrane that covered the dilated small and large bowel (arrow).

Intraoperatively, a thick fibrotic membrane encasing the entire small intestine and part of the large intestine was found. Obstruction was due to the pressure applied by the thick membrane on the small bowel loops. During the operation, the fibrotic membrane was excised, followed by adhesiolysis between the intestinal loops (Figure 5). The histological findings showed peritoneal fibrosis. The postoperative period was uncomplicated, and the patient was discharged on the 8^th^ postoperative day.

## 3. Discussion

ACS is a rare syndrome that mainly affects the small bowel, leading to intestinal obstruction. Although it was initially considered to be more frequent in young girls living in tropical and subtropical regions [1], a systematic review proved that it tends to be a male syndrome [3]. This is in agreement with our patients, who were middle-aged males.

The idiopathic form of ACS is extremely rare, whereas the secondary form is more common [15]. Clinically, the syndrome presents with acute or subacute small intestinal obstruction, with the involvement of the stomach, large intestine, liver, or other abdominal organs occurring infrequently [14]. The preoperative diagnosis of this syndrome is usually difficult. According to Yip and Lee, there are four main clinical features that allow for a preoperative diagnosis. The first feature is a bowel obstruction in a relatively young female patient without an obvious cause. The second one is a past history of similar episodes with spontaneous symptomatic relief. A presentation with symptoms suggestive of bowel obstruction, but with the absence of typical symptoms such as distention, is the third characteristic feature and the last one is the presence of a soft nontender abdominal mass [16]. Considering that these criteria have been in place for the last 25 years and that recent literature disputes them, it may be time for a revision.

An early preoperative diagnosis and treatment of this syndrome are vital to preserve the circulation of the encased bowel segments and reduce the risk of strangulation [17]. For this diagnosis, a CT scan is necessary as it can show peritoneal thickening, intestinal obstruction signs, and clustering and fixation of the intestinal loops [18]. According to literature, some cases (as in our first patient) are diagnosed radiographically as internal hernias [19]. However, the definite diagnosis of ACS is made during a laparotomy. The gold standard treatment is surgery that involves adhesiolysis and the partial or complete removal of the thick membrane. The histopathological findings should reveal intense peritoneal fibrosis with chronic nonspecific inflammation [20].

## 4. Conclusion

It is important for surgeons to be aware of ACS because the appropriate combination of clinical examination and imaging may facilitate the preoperative diagnosis.

## Abbreviations

ACS: Abdominal cocoon syndrome
CT: Computed tomography.
